# HATT: a phase IV, single-arm, open-label study of sorafenib in Taiwanese patients with advanced hepatocellular carcinoma

**DOI:** 10.1007/s12072-016-9774-x

**Published:** 2016-12-01

**Authors:** Shi-Ming Lin, Sheng-Nan Lu, Ping-Tsung Chen, Long-Bin Jeng, Shinn-Cherng Chen, Chi-Tan Hu, Sien-Sing Yang, Marie-Aude Le Berre, Xuan Liu, David Y. Mitchell, Klaas Prins, Joachim Grevel, Carol A. E. Peña, Gerold Meinhardt

**Affiliations:** 1 Division of Hepatology, Department of Gastroenterology and Hepatology, Linkou and Taipei Chang Gung Memorial Hospital Chang Gung University, No. 5 Fuhsing Street, Kuei Shan Hsiang, Taoyuan, Taiwan; 2 Division of Hepato-Gastroenterology, Kaohsiung Chang Gung Memorial Hospital, 100 Tapei Road, Niaosung, Kaohsiung, 833 Taiwan; 3 The Division of Hematology & Oncology, Chiayi Chang Gung Memorial Hospital, 6 W Sec, Chia Pu Road, Pu-Tz City, 613 Chiayi Taiwan; 4 Organ Transplantation Center, China Medical University Hospital, North District, Taichung City, 404 Taiwan; 5 Division of Hepato-Biliary-Pancreatic Medicine, Kaohsiung Medical University Hospital, No. 100, Tzyou 1st Road, Kaohsiung, 807 Taiwan; 6 Division of Hepato-Gastroenterology, Buddhist Tzu Chi General Hospital, Tzu Chi University, No. 707, Sec. 3 Chung Yang Road, Hualian City, 970 Hualien County Taiwan; 7 Liver Center, Cathay General Hospital, Taipei and Medical Faculty, Fu-Jen Catholic University, No. 280, Sec. 4 Jen-Ai Road, Taipei, 10630 Taiwan; 8 Clinical Statistics, Bayer HealthCare Pharmaceuticals, Medical Affairs, Building C2 1.11, 220 Ave de la Recherche, Loos, 59120 France; 9 Global Clinical Development, Bayer HealthCare Co. Ltd., 18F Tower B, Bayer Center, No. 27, Dong San Huan North Road, Chaoyang District, Beijing, China; 10 Mitchell Pharmaceutical Consulting, 1188 Hawk Ridge Road, Lafayette, CO 80026 USA; 11 qPharmetra, LLC, Kwakkenbergweg 39, 6523 MK Nijmegen, The Netherlands; 12 BAST Inc Limited, 7 Wellingtonia Close, Shepshed, LE12 9FB UK; 13 Clinical Sciences, Bayer Healthcare Pharmaceuticals, 100 Bayer Road, Whippany, NJ USA; 14 Global Clinical Development Oncology, Bayer Healthcare Pharmaceuticals, 100 Bayer Road, Whippany, NJ USA

**Keywords:** Sorafenib, Advanced hepatocellular carcinoma, Metastatic hepatocellular carcinoma, Taiwanese patients, Overall survival, Time to progression

## Abstract

**Background:**

Sorafenib significantly improves survival in patients with advanced hepatocellular carcinoma (HCC). This phase IV study assessed sorafenib efficacy/safety in Taiwanese patients with advanced HCC and Child–Pugh A status.

**Methods:**

All patients received 400 mg sorafenib BID. Safety, efficacy, sorafenib pharmacokinetics, and Child–Pugh progression were evaluated. A hand-foot skin reaction (HFSR) prevention substudy assessed HFSR incidence and grade/severity and time to HFSR in 29 and 34 patients randomized to corticosteroid and noncorticosteroid ointments, respectively, and in 88 nonrandomized patients.

**Results:**

The 151 patients included 120 (80%) male patients and 81 (54%) with stage IV disease. Mean sorafenib dose was 626 mg/day, and median treatment duration was 4.2 months. Median overall survival (OS), progression-free survival, and time to progression (TTP) were 8.6, 2.7, and 3.8 months, respectively. Disease control and response rates (partial responses only) were 48 and 6.6%, respectively. Median TTP from Child–Pugh A to B/C was 88 days. Drug-related adverse events (AEs) occurred in 89.4% of patients; none were new or unexpected. The most frequent grade ≥3 drug-related, treatment-emergent AEs were HFSR (13.2%), diarrhea (11.9%), and hypertension (6.6%). Corticosteroid ointment tended to reduce the severity and incidence of all HFSR-associated parameters. Pharmacokinetic exposure was unaltered by Child–Pugh progression. The final pharmacokinetic model predicted 13.1 and 33.8% reductions in sorafenib exposure over 6 and 12 months, respectively.

**Conclusions:**

There was a trend of longer OS and TTP in Taiwanese patients with advanced HCC compared with patients with advanced HCC in the Asia–Pacific trial. Sorafenib exposure did not correlate with liver function. Reduced pharmacokinetic exposure over time was unrelated to reduced or interrupted dosing.

**Electronic supplementary material:**

The online version of this article (doi:10.1007/s12072-016-9774-x) contains supplementary material, which is available to authorized users.

## Introduction

Hepatocellular carcinoma (HCC) is the sixth most common cancer worldwide and the third most common in the Asia–Pacific region [1, 2]. Geographical differences in HCC incidence are largely due to variations in hepatitis B and C infection [3, 4]. In East Asia, including Taiwan, where hepatitis B virus (HBV) is endemic, the incidence rate of HCC is 20–28 per 100,000 people. Approximately 10–20% of cases of HCC occur in patients with chronic HBV infection in the absence of cirrhosis.

Two randomized, placebo-controlled, phase III trials, the Sorafenib HCC Assessment Randomized Protocol (SHARP) and the Sorafenib Asia–Pacific (Sorafenib AP) trials, showed that the multikinase inhibitor sorafenib significantly improves overall survival (OS) and progression-free survival (PFS) in patients with advanced HCC [5, 6]. Based on these results, sorafenib was approved as systemic treatment for patients with advanced HCC and remains the only globally approved systemic treatment for this disease.

As a post-approval commitment in Taiwan, 151 patients were enrolled in this phase IV, single-arm study [Hepatocellular carcinoma–Advanced stage–sorafenib Trial in Taiwanese patients (HATT)] to confirm the efficacy and safety of sorafenib. The requested number of patients was based on the number of patients from the mainland of China who were treated in the phase 3 Asia–Pacific study (NCT00492752), which was 151 of the 226 randomized patients [5]. The main objective of the post-authorization study was to evaluate the safety and efficacy profile of sorafenib and to evaluate Child–Pugh status progression in Taiwanese patients with advanced HCC treated with sorafenib. The main study did not have a primary endpoint. Another objective of this main study was to assess the pharmacokinetics of sorafenib in patients with HCC. A substudy of hand-foot skin reaction (HFSR) prevention assessed HFSR incidence and grade/severity and time to HFSR in patients randomized to corticosteroid and noncorticosteroid ointment and in a group of nonrandomized, untreated patients.

## Methods

This prospective, open-label, single-arm, post-authorization study was conducted across seven sites in Taiwan in patients with advanced HCC. All patients meeting entry criteria received sorafenib 400 mg (2 × 200-mg tablets) twice daily (BID) on a continuous schedule. For the purpose of data recording, treatment period was divided into 21-day cycles. Treatment continued beyond radiologic progression, provided the subject derived clinical benefit, as judged by the treating physician.

### Ethical considerations

The trial protocol was approved by the institutional review boards of all seven institutions. All patients provided written informed consent. The trial has been registered at clinicaltrials.gov as NCT01098760.

### Inclusion criteria

Patients aged ≥18 years were included if they had cytologically or histologically documented, unresectable advanced and/or metastatic HCC not amenable to local treatment methods, with at least one tumor lesion that could be measured accurately in at least one dimension and had not been treated with local therapy. HCC in cirrhotic patients was diagnosed by American Association for the Study of Liver Disease criteria; noncirrhotic patients required histological or cytologic confirmation. Patients who previously received local therapy (e.g., surgery, radiation, hepatic artery embolization, transarterial chemoembolization, radiofrequency ablation, percutaneous ethanol injection, or cryoablation) were deemed eligible, but local therapy had to be completed at least 4 weeks before study entry, and all toxic effects of any prior local treatments had to have resolved. Moreover, previously treated lesions could not be selected as target lesions. Other inclusion criteria included Child–Pugh Class A, Eastern Cooperative Oncology Group performance status of 0–2, and life expectancy ≥ 12 weeks. The first patient’s first visit was in August 2010 and the last patient’s first visit was in June 2012.

### Exclusion criteria

Patients were excluded if they had previous or concurrent cancer, unless curatively treated >3 years before study entry; renal failure requiring dialysis; a history of cardiac disease, including congestive heart failure, active coronary artery disease, or cardiac arrhythmias; or uncontrolled hypertension (systolic blood pressure >150 mmHg or diastolic blood pressure >90 mmHg). Also excluded were patients with active, clinically serious infections [except for HBV/hepatitis C virus (HCV)]; central nervous system tumors; clinically significant gastrointestinal bleeding <30 days before study entry; history of organ allograft transplantation; Child–Pugh Class B or C liver status; or uncontrolled ascites. Patients previously treated with yttrium-90 spheres, those with clinically significant peripheral vascular disease or a history of substance abuse or psychological conditions interfering with participation, and patients unable to take oral medications were also excluded.

### Dose modifications

All patients were initially treated with 400 mg sorafenib BID. Reductions to 400 mg once daily and 400 mg every other day (QOD) were permitted for clinically significant hematologic and nonhematologic toxicities. Treatment was decreased one dose level for grade 3 hematologic toxicities. Treatment was interrupted in patients with grade 4 hematologic toxicities and in patients with grade 3 nonhematologic toxicities (except skin toxicity and hypertension) until they achieved grade ≤2 with treatment resumed at one lower dose level. Treatment was also discontinued in patients with grade 4 nonhematologic toxicities and in patients who would have required further dose reductions beyond 400 mg QOD. In patients who experienced two or more toxicities, reductions were according to the toxicity with the highest grade; alternatively, if both toxicities were of equal grade, dose was reduced according to the toxicity deemed most causally related to study treatment. Increases were permitted in patients who previously had dose reductions and remained on stable doses for ≥3 weeks without further toxicities, although only one such increase per patient was permitted.

### Safety and efficacy assessments

Safety was evaluated at screening (within 28 days of first dose of study drug), before dosing on the first day of study drug administration, and before dosing every 3 weeks (±7 days). Tumors were assessed within 28 days of the start of study drug using CT or MRI scans. For the purpose of data recording, the treatment period was divided into 3-week cycles. Tumor measurements and evaluation of tumor response were performed within the last 7 days of every other treatment cycle, beginning with the second cycle. Child–Pugh status was determined at screening and on day 1 of each cycle.

### Follow-up

After completion of study drug treatment, patients were contacted every 3 months to determine survival status. Follow-up was continued until 6 months after the first treatment of the last patient. For regulatory purposes, the end of the trial was defined as 6 months after the first treatment of the last patient but not before 106 patients had died.

### Pharmacokinetic analysis

After enrollment started, the study protocol was amended to include a steady-state pharmacokinetic analysis, as requested by the Taiwan Department of Health. Sorafenib pharmacokinetics were assessed in a subpopulation of 85 patients. Blood samples were collected on day 1 of each 21-day cycle, starting with cycle 2, to assess sorafenib exposure concurrently with each determination of Child–Pugh status. Sorafenib concentrations in plasma were determined by high-pressure liquid chromatography and tandem mass spectrometry. Summary statistics were generated describing sorafenib plasma concentrations of all pharmacokinetics samples at a given Child–Pugh score.

Plasma sorafenib concentrations were analyzed using a previously defined population pharmacokinetic model [7, 8] and the software program, NONMEM v.7.2 (ICON, Dublin, Ireland). Briefly, the previously developed model applied to the HATT data used a 1-compartment model with three sequential transit absorption compartments and included sex, body weight, body mass index, race (Asian/nonAsian), Child–Pugh score, age, liver transaminases, albumin, bilirubin, alkaline phosphatase, lactate dehydrogenase, serum glutamic oxaloacetic transaminase, serum glutamic pyruvic transaminase, prothrombin time, creatinine clearance, co-medication with CYP3A4 or UGT1A9 inducers or inhibitors, or thyroxine as a covariate for sorafenib apparent clearance (CL/F). For the current analyses, gender was the only covariate included in the model because it was the one covariate that significantly influenced CL/F in the meta-analysis that was performed to develop the population pharmacokinetic model. Predicted sorafenib concentrations were calculated based on daily doses and dose reductions, interruptions, and discontinuations. Observed and predicted sorafenib concentrations from the final model were compared, as were changes over time.

### Incidence of and time to hand-foot skin reaction

During the HATT trial, the study protocol was amended to include assessment of the impact of two different ointments on HFSR prevention. Beginning at the start of treatment, 29 patients were randomized to a corticosteroid and 34 to a noncorticosteroid ointment, to be applied twice daily to soles and palms for 21 days. The rates of HFSR after 3 and 6 weeks and overall, as well as time to HFSR and grade/severity of HFSR, were compared in these two groups and with a group of 88 patients enrolled before the start of the HFSR prevention substudy. Exposure to sorafenib was also assessed in these three subgroups, as measured by duration of treatment, total dose, and number of dose modifications.

### Statistical analysis

Demographic variables and baseline characteristics were summarized descriptively for all patients in the intent-to-treat population. Continuous variables were reported as mean ± standard deviation or medium (minimum, maximum), and categorical variables were reported as frequency (%). Time to event variables, including OS, PFS, time to progression (TTP), and time from Child–Pugh A to Child–Pugh B/C, were assessed by the Kaplan–Meier method and compared by the log-rank test. Treatment-emergent adverse events (AEs), drug-related AEs, AEs leading to premature termination, dose reductions and interruptions, serious AEs (SAEs), and laboratory parameters were summarized using descriptive statistics for safety.

## Results

### Patient characteristics and exposure to sorafenib

**Fig. 1:**
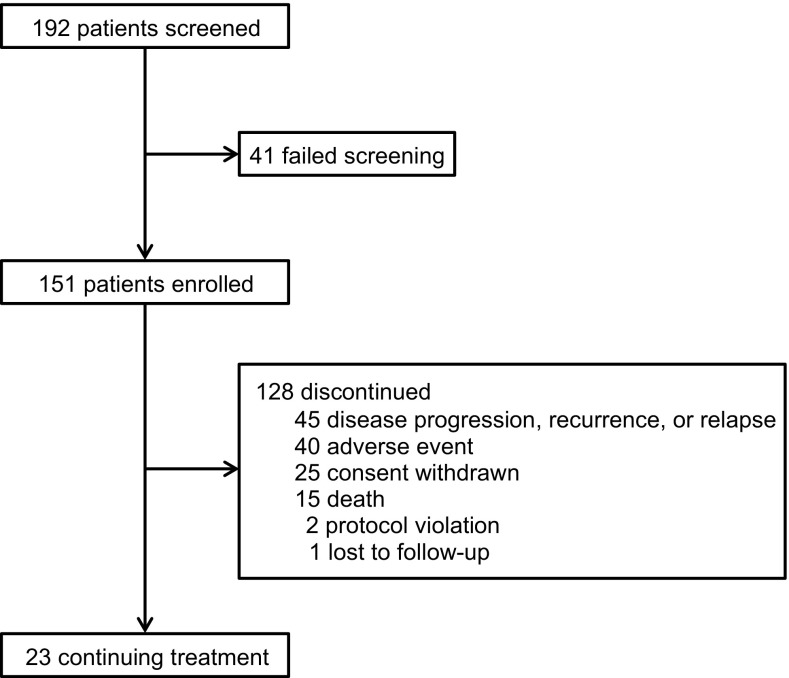
CONSORT diagram

Most patients (54%) had stage IV disease at study entry (Table 1). The primary causes of HCC in these patients were HBV infection (*n* = 81, 53.6%) and HCV infection (*n* = 41, 27.2%; Table 1). Of the 151 patients, 23 (15.3%) were continuing treatment at the time of database cutoff, whereas 128 (84.8%) terminated treatment, most for AEs, disease progression/recurrence, and withdrawal of consent (Fig. 1).

**Table 1:** Demographic characteristics and disease history of Taiwanese patients with HCC

Characteristics	Total patients (*n* = 151)
Median age, year (range)	62.0 (28–97)
Male patients, *n* (%)	120 (79.5)
Mean ± SD weight, kg	62.35 ± 10.55
Mean ± SD BMI, kg/m^2^	23.58 ± 3.26
Etiology, *n* (%)	
Hepatitis B	81 (53.6)
Hepatitis C	41 (27.2)
Alcohol use	6 (4.0)
Hepatitis B and C	5 (3.3)
Hepatitis B and alcohol	3 (2.0)
Nonalcoholic steatohepatitis	2 (1.3)
Hepatitis C and alcohol	1 (0.7)
Hepatitis B and C and alcohol	1 (0.7)
Unknown	11 (7.3)
ECOG PS, *n* (%)	
0	123 (81.5)
1	27 (17.9)
2	1 (0.7)
Child–Pugh classification, *n* (%)	
A	149 (98.7)
B	2 (1.3)
Child–Pugh Score, *n* (%)	
5	80 (53.0)
6	69 (45.7)
7	1 (0.7)
8	1 (0.7)
Disease stage, *n* (%)	
II	8 (5.3)
IIIA	32 (21.2)
IIIB	17 (11.3)
IIIC	13 (8.6)
IV	81 (53.6)
Liver cirrhosis	144 (95.4)
AFP >200 ng/mL	99 (65.6)

*AFP* alpha-fetoprotein, *BMI* body mass index, *ECOG PS* Eastern Cooperative Oncology Group performance score, *HCC* hepatocellular carcinoma, *SD* standard deviation

Although the study protocol required that all patients be Child–Pugh class A, two (1.3%) were Child–Pugh class B at baseline. Protocol deviations were documented for both patients.

Assessment of time of patient exposure to sorafenib showed that the median duration of treatment was 18.1 weeks (range, 0.3–124 weeks), and the mean ± SD duration of treatment was 29.9 ± 30.3 weeks. During this time, patients received a median 661.5 mg/day (range, 211.5–800 mg/day) and a mean ± SD of 625.9 ± 170.3 mg/day sorafenib. Of the 151 patients, 112 (74.2%) required dose reductions, and 99 (65.8%) required dose interruptions.

### Efficacy

**Fig. 2:**
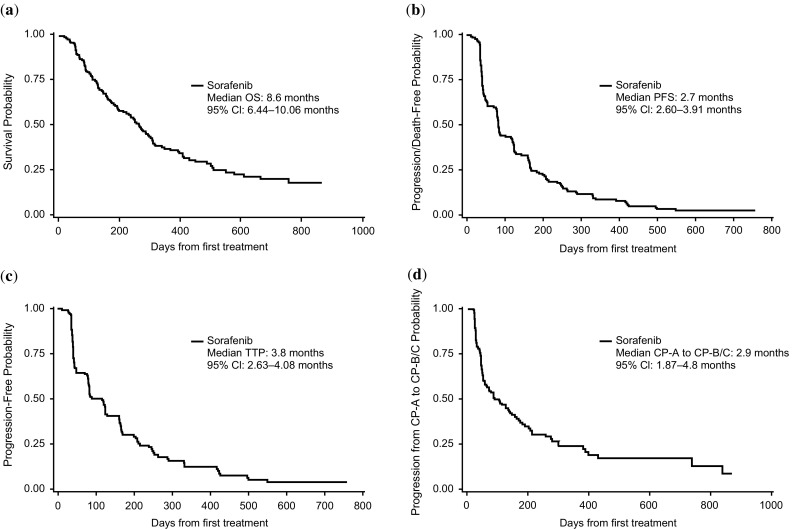
Efficacy outcomes. Kaplan–Meier plots of **a** OS, **b** PFS, **c** TTP, and **d** time from Child–Pugh A to Child–Pugh B/C liver status in patients enrolled in the HATT trial. *OS* overall survival, *PFS* progression-free survival, *TTP* time to progression, *HATT* Hepatocellular Carcinoma–Advanced Stage–Sorafenib Trial in Taiwanese patients

Median OS was 8.6 months (95% CI, 6.4–10.1 months); median PFS was 2.7 months (95% CI, 2.6–3.9 months); and median TTP was 3.8 months (95% CI, 2.6–4.1 months; Fig. 2).

Ten patients (6.6%) showed a partial response to treatment, and 62 (41.1%) had confirmed stable disease. Thus, the overall response rate was 6.6% and the disease control rate was 47.7%. Descriptive analysis of mean alpha fetoprotein (AFP) levels showed that AFP levels were variable but decreased from baseline by treatment cycle 6 (Supplemental Fig. 1). At the end of treatment, 73 patients were classified as Child–Pugh A, 50 as Child–Pugh B, and 12 as Child–Pugh C. The median time to worsening of liver function, from Child–Pugh A to first detection of Child–Pugh B or C, was 2.9 months (95% CI, 1.9–4.8 months) (Fig. 2). Child–Pugh scores of individual patients did not consistently progress over time, from A to B, B to C, and A to C; rather, Child–Pugh scores varied within a patient between visits, with some patients showing improvements after initial worsening and others showing progressive deterioration.

### Safety

There were no unexpected AEs. Drug-related treatment-emergent AEs were reported in 135 patients (89.4%), with grade 1/2 drug-related AEs occurring in 63 patients (41.7%) and grade 3/4 drug-related AEs occurring in 72 patients (47.7%) (Table 2). The most frequently reported drug-related, treatment-emergent AEs in this trial were HFSR (64.9%), diarrhea (45.0%), and ascites (27.2%), whereas the most frequently reported grade 3 drug-related, treatment-emergent AEs were HFSR (13.2%), diarrhea (11.9%), and hypertension (6.6%). Treatment-emergent SAEs were reported in 110 patients (72.8%), and drug-related treatment-emergent SAEs in 14 (9.3%). Grades 3 and 4 drug-related, treatment-emergent SAEs were reported in 10 (6.6%) and 3 (2.0%) patients, respectively, but there were no drug-related, treatment-emergent grade 5 SAEs.

**Table 2:** Percentage of patients with drug-related, treatment-emergent AEs observed in >5% of patients

AE	Total	%Grade 3/4
HFSR	64.9	13.2/0
Diarrhea	45.0	11.9/0
Alopecia	27.2	0/0
Hypertension	18.5	6.6/0
Fatigue	11.3	0.7/0
Rash maculopapular	11.3	0.7/0
Platelet count decreased	9.9	4.0/1.3
Pruritus	8.6	0.7/0
AST increased	6.6	4.0/1.3
Anorexia	6.0	0/0
Hoarseness	5.3	0/0
Weight loss	5.3	0/0

*AEs* adverse events, *AST* aspartate aminotransferase, *HFSR* hand-foot skin reaction

### Pharmacokinetics

**Fig. 3:**
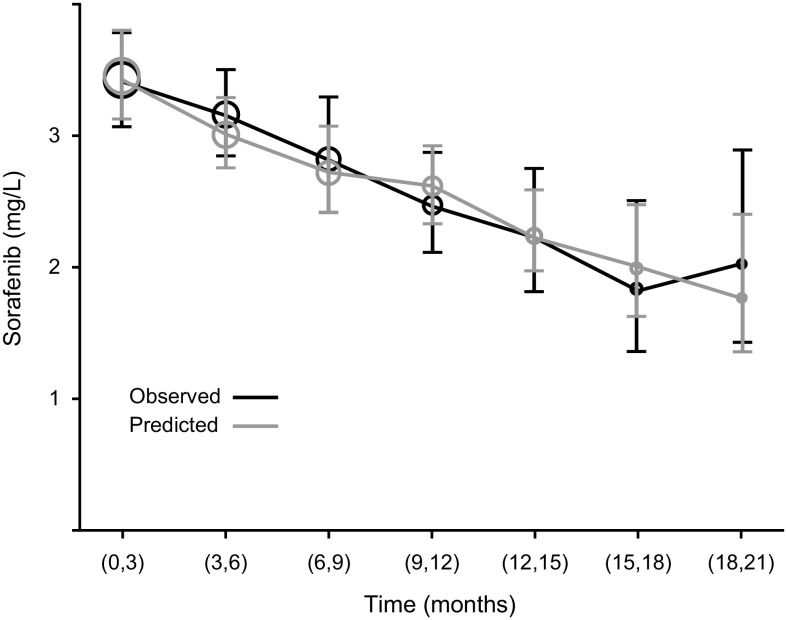
Observed and predicted sorafenib concentrations over time. The final pharmacokinetics model predicted reductions in sorafenib exposure of 13.1 and 33.8% over 6 and 12 months of treatment, respectively, as shown by comparing the 3- to 6-month interval with the 9- to 12- and 15- to 18-month intervals. Time was stratified by quarter, with predicted concentrations representing individual values. Data are presented as geometric means and 95% confidence intervals

Of the 151 patients, 85 (56.3%) provided a total of 847 plasma samples for pharmacokinetic analysis. Median sorafenib concentrations at the time of pharmacokinetic sampling of patients classified as Child–Pugh A, B, and C were 3.72 mg/L (range, 0.0162–15.6 mg/L), 2.90 mg/L (range, 0.0124–15.6 mg/L), and 3.29 mg/L (range, 0.0287–7.44 mg/L), respectively (Supplemental Fig. 2). Application to the HATT trial of a population pharmacokinetic model developed from 10 phase I to III trials of sorafenib in healthy volunteers and in patients with renal cell carcinoma, HCC, differentiated thyroid cancer, and other tumor types, including patient sex as a covariate with sorafenib CL/F [8], suggested a trend toward decreasing sorafenib concentrations over time that was not accounted for by the model (Supplemental Fig. 3). Examination of sex as a covariate indicated a 40.5% lower overall CL/F in female than in male patients. Linear and power relationships for a change in clearance or bioavailability over time were tested, with the power model providing the best fit. Inclusion of albumin and bilirubin as covariates improved the model fit, whereas alkaline phosphatase, prothrombin time, international normalized ratio, serum glutamic oxaloacetic transaminase, serum glutamic pyruvic transaminase, and Child–Pugh score did not affect sorafenib exposure. Both bilirubin and albumin concentrations were positively correlated with sorafenib concentrations, although only bilirubin showed a time dependence, with sorafenib concentrations and bilirubin levels decreasing together over time. Using the final population pharmacokinetic model, the observed and predicted plasma sorafenib concentrations in individual patients demonstrated concordance over the entire time range studied (Table 3, Fig. 3). The final pharmacokinetic model predicted reductions of 13.1% and 33.8% in sorafenib exposure over 6 and 12 months, respectively.

**Table 3:** Observed and predicted sorafenib exposure in patients over time

Type	Time (months)	*n*_obs_	*n*_ind_	Geometric mean (mg/L)	95% CI	Diff (%)
Observed	0–3	332	85	3.42	3.07–3.81	8.42
	3–6	188	55	3.15	2.83–3.51	0
	6–9	125	35	2.81	2.40–3.33	−10.7
	9–12	95	25	2.47	2.11–2.88	−21.7
	12–15	56	18	2.23	1.80–2.76	−29.4
	15–18	31	9	1.84	1.34–2.52	−41.7
	18–21	20	6	2.02	1.40–2.91	−36.0
Predicted	0–3	332	85	3.46	3.14–3.81	14.7
	3–6	188	55	3.01	2.76–3.30	0
	6–9	125	35	2.72	2.41–3.08	−9.62
	9–12	95	25	2.62	2.33–2.94	−13.1
	12–15	56	18	2.26	1.97–2.60	−25.0
	15–18	31	9	2.00	1.61–2.48	−33.8
	18–21	20	6	1.79	1.34–2.40	−40.6

*Diff (%)* the percentage difference in mean sorafenib exposure relative to the mean sorafenib exposure in the 3- to 6-month period

*CI* confidence interval, *n*_*obs*_ number of observations, *n*_*ind*_ number of individuals

### Hand-foot skin reaction substudy

**Fig. 4:**
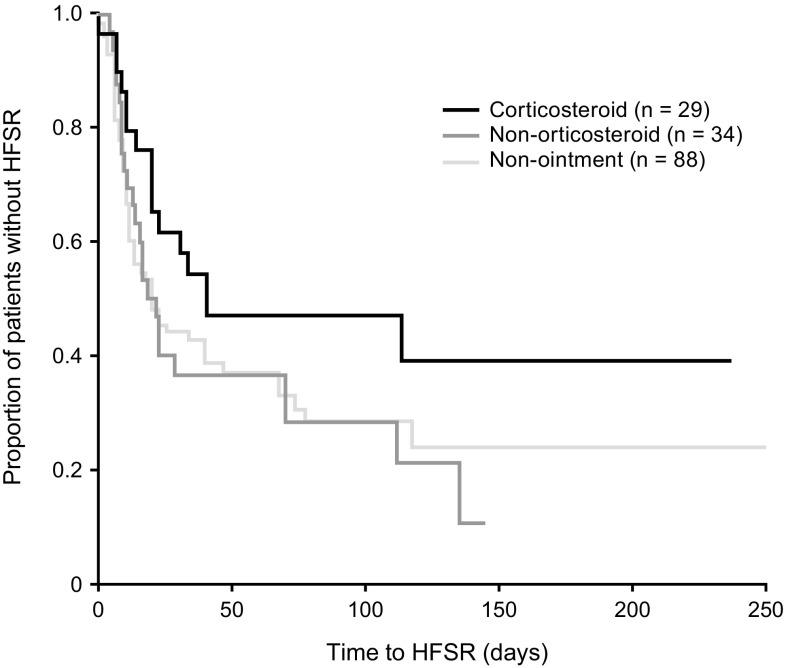
Kaplan–Meier analysis of time to HFSR in sorafenib-treated HCC patients randomized to corticosteroid (*n* = 29) and noncorticosteroid (*n* = 34) ointments and in nonointment-treated patients from the HATT trial registered before the beginning of the HFSR prevention substudy (*n* = 88). *HFSR* hand-foot skin reaction, *HCC* hepatocellular carcinoma, *HATT* Hepatocellular Carcinoma–Advanced Stage–Sorafenib Trial in Taiwanese patients

The incidence of overall HFSR and at weeks 3 and 6 did not differ significantly between patients randomized to corticosteroid and noncorticosteroid ointments (Table 4). HFSR incidence rates were also not significantly different between patients randomized to corticosteroid cream and those not treated with ointment (Table 4). HFSR scores at 3 and 6 weeks did not differ significantly between patients randomized to corticosteroid and noncorticosteroid treatment, and between patients randomized to corticosteroid treatment and those not treated with ointment (Table 4). However, overall HFSR scores were significantly different between patients randomized to corticosteroid cream and noncorticosteroid ointments (0.83 vs. 1.26, *p* = 0.031) and between patients randomized to corticosteroid cream and those not treated with ointment (0.83 vs. 1.24, *p* = 0.038) (Table 4). Time to HFSR was longer in the group treated with corticosteroid cream (41 days) than in the group treated with noncorticosteroid cream (22 days) and the untreated control group (21 days), although these differences were not statistically significant. The incidence of grade 3 HFSR was low in both arms of the substudy (5.9% in the noncorticosteroid group and 0% in the corticosteroid group; no grade 4 or 5 events were reported). However, the incidence of grade 3 HFSR (20.5%) was higher in the nonointment group (Table 4). Corticosteroid treatment tended to reduce the severity and incidence of all HFSR-associated parameters (Fig. 4).

**Table 4:** Incidence of grade 3–5 treatment-emergent AEs reported for >10% of patients in any study arm and incidence of HFSR and HFSR scores in patients treated with corticosteroid and noncorticosteroid ointments and in nonointment-treated patients from the HATT trial registered prior to the beginning of the HFSR prevention substudy

	Randomized into substudy			Nonrandomized	
	Corticosteroid group (*n* = 29)	Non-corticosteroid group (*n* = 34)		Nonointment group (*n* = 88)	
TEAE, *n* (%)					
Anemia	2 (6.9)	4 (11.8)		11 (12.5)	
Abdominal pain	1 (3.4)	3 (8.8)		13 (14.8)	
Ascites	3 (10.3)	2 (6.1)		13 (14.8)	
Diarrhea^a^	3 (10.3)	5 (14.7)		11 (12.5)	
Hepatic failure	1 (3.4)	1 (2.9)		12 (13.6)	
Elevated alanine aminotransferase	3 (10.3)	2 (5.9)		12 (13.6)	
Elevated aspartate aminotransferase	4 (13.8)	5 (14.7)		24 (27.3)	
Elevated blood bilirubin	3 (10.3)	4 (11.8)		19 (21.6)	
Decreased platelet count	2 (6.9)	4 (11.8)		11 (12.5)	
Hyponatremia	2 (6.9)	7 (20.6)		12 (13.6)	
Hypophosphatemia^a^	3 (10.3)	1 (2.9)		4 (4.5)	
Encephalopathy	1 (3.4)	4 (11.8)		12 (13.6)	
HFSR^a^	0	2 (5.9)		18 (20.5)	
Hypertension^a^	5 (17.2)	5 (14.7)		9 (10.2)	

			*p* value^b^		*p* value^c^
HFSR, *n* (%)					
3 weeks	10 (34.5)	16 (47.1)	0.1561	44 (50.0)	0.0730
6 weeks	15 (51.7)	20 (58.8)	0.2860	52 (59.1)	0.2434
Overall	16 (55.2)	24 (70.6)	0.1026	58 (65.9)	0.1492
Grade 3 HFSR^a^, *n* (%)					
3 weeks	0 (0)	1 (2.9)	N.D.	11 (12.5)	N.D.
6 weeks	0 (0)	2 (5.9)	N.D.	13 (14.8)	N.D.
Overall	0 (0)	2 (5.9)	N.D.	18 (20.5)	N.D.
HFSR score					
3 weeks	0.55	0.74	0.2026	0.86	0.0744
6 weeks	0.72	1.00	0.1159	1.03	0.0784
Overall	0.83	1.26	0.0314	1.24	0.0380
Median time to HFSR onset, *d* (range)	41 (1–238)	22 (5–145)	0.0639	21	0.0782

All *p* values are 1-sided

*HFSR* hand-foot skin reaction, *N.D.* not determined, *TEAE* treatment-emergent adverse events

^a^No grade 4 or 5 AEs were reported

^b^Comparisons of patients randomized to corticosteroid cream and noncorticosteroid ointments

^c^Comparisons of patients randomized to corticosteroid cream and those not treated with ointment

## Discussion

As a post-approval commitment, 151 patients from Taiwan were enrolled in this phase IV, single-arm study to confirm the efficacy and safety of 400 mg BID sorafenib in patients with advanced HCC, including patients with metastatic disease, those deemed unresectable and ineligible for local treatment, and those with local treatment failure. The results for OS, PFS, and TTP mirrored or exceeded those observed in the Sorafenib AP trial. For example, patients in this trial had a median OS of 8.6 months (95% CI, 6.44–10.06 months) and a median TTP of 3.8 months (95% CI, 2.63–4.08 months). In comparison, sorafenib-treated patients in the AP trial had a median OS of 6.5 months (95% CI, 5.56–7.56 months) and a median TTP of 2.8 months (95% CI, 2.63–3.58 months) [5]. However, median OS and TTP in this trial were in line with the sorafenib control arms of Asian patients in recent phase III trials investigating sunitinib and brivanib in advanced HCC [9, 10]. The trend toward longer survival of patients with advanced HCC enrolled in current compared with earlier clinical trials may be due to better disease characteristics at enrollment and more physician experience handling the AEs associated with sorafenib.

Patients with advanced HCC are prone to deterioration of liver function, largely due to worsening of underlying cirrhosis and/or progression of intrahepatic tumor lesions, but in some patients the cause is drug-induced toxicity. The median time for progression from Child–Pugh A to Child–Pugh B or C was 2.9 months (95% CI, 1.87–4.8 months). Nevertheless, median sorafenib concentrations in patients progressing to Child–Pugh B or C were similar to those in patients with Child–Pugh A, suggesting that sorafenib exposure was not associated with Child–Pugh status. A small number of patients (*n* = 12) had Child–Pugh C scores at the end of treatment. Although patient-level data on causes for change from Child–Pugh A to Child–Pugh C score were not collected, most patients who discontinued sorafenib therapy had disease progression. Disease progression may be a key contributor to the change from Child–Pugh A to Child–Pugh C, although a separate prospective study is needed to determine predictors of change in Child–Pugh score in patients on sorafenib treatment. Of note, consistent progression over time in Child–Pugh scores (i.e., from A to B, B to C, and A to C) was not observed in all patients. Instead, in some patients, Child–Pugh scores varied within patients over time, with some patients showing improvements after initial worsening and others demonstrating progressive deterioration.

Application of a population pharmacokinetic model suggested that sorafenib concentrations tended to decrease over time. Concentrations of albumin and bilirubin were positively correlated with sorafenib concentrations, although only bilirubin concentration showed a time dependence, possibly due to sorafenib inhibiting metabolism of bilirubin by UGT1A1 [11]. The final pharmacokinetic model predicted decreases in sorafenib exposure over 6 and 12 months of 13.1 and 33.8%, respectively. This change in concentration was not associated with reductions in liver function, as shown by Child–Pugh score, nor was it due to reductions or interruptions in sorafenib dosing. The mechanisms underlying these reductions in sorafenib exposure, and the clinical impact of these reductions, have not yet been determined. Nonlinear mixed effects modeling using NONMEM is based on datasets containing the actual doses taken and, should they occur, naturally adjusts the model predictions for dosing changes. Because the model predictions insufficiently accounted for reductions in observed concentrations, the changes are therefore caused by factors other than dosing reductions/interruptions.

### Hand-foot skin reaction

HFSR is a frequent AE of tyrosine kinase inhibitors, including sorafenib, that target the vascular endothelial growth factor signaling pathway [5, 6, 12–16]. Topical steroids have been reported effective in the treatment of sorafenib-induced HFSR [17–19]. Analysis of incidence, time to HFSR, and HFSR score in a subset of patients in this study randomized to a corticosteroid or a noncorticosteroid cream found that those randomized to the corticosteroid cream tended to have a longer time to onset of HFSR and a better HFSR score than patients randomized to the noncorticosteroid cream, although most differences were not statistically significant. Interestingly, although the initial cohort of patients not prescribed ointment before the randomized HFSR prevention substudy had similar HFSR incidence, time to HFSR, and HFSR scores as patients randomized to noncorticosteroid cream, the incidence of grade 3 HFSR was noticeably lower in the noncorticosteroid cream group than in the nonointment group (i.e., the initial 88 nonrandomized patients), potentially supporting the value of any type of ointment for HFSR prophylaxis. A recent trial involving 871 Chinese patients treated for HCC found that prophylactic administration of a urea-based cream significantly reduced the 12-week incidence of any grade HFSR (56.0% vs. 73.6%; OR, 0.457; 95% CI, 0.344–0.608; *p* < 0.0001) and of grade ≥ 2 HFSR (20.7% vs. 29.2%%; OR, 0.635; 95% CI, 0.466–0.866; *p* = 0.004) compared with best-supportive care alone [19]. Moreover, the median time to first occurrence of HFSR was significantly longer in the urea-based cream than in the control group (84 vs. 34 days; hazard ratio, 0.658; 95% CI, 0.541–0.799; *p* < 0.0001). Taken together, these findings suggest that prophylactic treatment with a urea-based or corticosteroid-containing cream may benefit patients being treated with sorafenib for HCC.

Although, in the main study, AEs such as HFSR, ascites, and diarrhea were frequent, all were manageable and rarely resulted in discontinuation from treatment. There were no new or unexpected safety findings.

In conclusion, the outcome of this study confirms the results of the previous Sorafenib AP study. The pharmacokinetic model predicted decreases in sorafenib exposure, but the clinical significance of the observed 33.8% decrease in exposure after 1 year remains unclear, especially given the shorter median and mean treatment duration in this patient population. Sorafenib remains the standard of care for Asian patients with advanced/metastatic HCC.

## Electronic supplementary material

Below is the link to the electronic supplementary material.

Supplemental Fig1 Mean AFP levels over time. AFP was measured during the screening period, at even cycles during treatment, and at the end of treatment visit (after cessation of treatment). Timepoints with ≥10 patients represented are shown. *AFP* alpha fetoprotein (EPS 1434 kb)

Supplemental Fig2 *Box*-and-*whisker plots* of sorafenib plasma concentration by Child–Pugh score (EPS 566 kb)

Supplementary material 3 (EPS 2057 kb)

Supplementary material 4 (EPS 781 kb)

Supplemental Fig3 **a** Predicted concentrations of sorafenib over time, based on a power model developed in healthy volunteers and in patients with renal cell carcinoma, HCC, differentiated thyroid cancer, and other tumor types. **b**, **c** Actual concentrations of sorafenib over time in patients enrolled in the pharmacokinetic subpopulation of the HATT trial showing (**b**) patients who discontinued treatment in <1 year and (**c**) patients who remained in the study for >1 year (EPS 861 kb)

Supplementary material 6 (EPS 2057 kb)

Supplementary material 7 (EPS 781 kb)

Supplementary material 8 (EPS 861 kb)
